# Whey protein supplementation for the preservation of mass and muscular strength of patients with heart failure: study protocol for a randomized controlled trial

**DOI:** 10.1186/s13063-018-2811-4

**Published:** 2018-08-08

**Authors:** Elisa Maia dos Santos, Roger de Moraes, Eduardo Vera Tibiriça, Grazielle Vilas Bôas Huguenin, Annie Seixas Belo Moreira, Andrea Rocha De Lorenzo

**Affiliations:** 1 Institute of the Heart Edson Saad, Federal University of Rio de Janeiro (UFRJ), Rio de Janeiro, RJ Brazil; 2 Universidade Estácio de Sá, Rio de Janeiro, RJ Brazil; 3 Laboratory of Cardiovascular Investigation, Oswaldo Cruz Institute (IOC), Rio de Janeiro, RJ Brazil; 4 Universidade Federal Fluminense (UFF), Niterói, Brazil; 5 National Institute of Cardiology (INC), Rio de Janeiro, RJ Brazil; 6 State University of Rio de Janeiro, Rio de Janeiro, RJ Brazil; 7 Department of Clinical Research, National Institute of Cardiology (INC), Rua das Laranjeiras, 374, 5o andar – Laranjeiras, Rio de Janeiro, RJ CEP: 22240-006 Brazil

**Keywords:** Heart failure, Cardiac rehabilitation, Randomized controlled trial, Study design, Whey proteins

## Abstract

**Background:**

Heart failure (HF) is an important public health problem, considered a new epidemic with high morbidity and mortality. The progression of HF often determines weight reduction, muscle mass loss, and reduced physical ability. Whey protein supplementation may increase the effects of exercise on strength and muscle mass, in addition to promoting improved endothelial function, body composition and quality of life. However, studies are needed to evaluate its benefits in patients with HF.

**Methods/design:**

This is a double-blind, randomized, placebo-controlled clinical trial in which patients with HF will be randomly allocated to two groups to receive supplementation with whey protein or placebo, associated with supervised exercise, for 12 weeks. The frequency of exercise will be three times a week. The study variables will be evaluated at baseline and 12 weeks. The main outcome will be maintenance of muscle mass and strength. Microvascular reactivity, quality of life, and inflammatory parameters will be evaluated as secondary outcomes.

**Discussion:**

HF is associated with severe loss of muscle mass and strength, directly contributing to exercise intolerance and inability to maintain daily life activities, becoming a strong predictor of reduced quality of life and mortality. The results of this study will add to the evidence base for providing new dietary recommendations.

**Trial registration:**

ClinicalTrials.gov, ID: NCT03142399. Registered on 29 May 2016. Effect of Whey Protein’ Supplementation and Exercise in Patients with Heart Failure (PROT-HF).

**Electronic supplementary material:**

The online version of this article (10.1186/s13063-018-2811-4) contains supplementary material, which is available to authorized users.

## Background

Heart failure (HF) is a clinical syndrome associated with poor quality of life, high utilization of economic resources, largely related to high hospitalization rates, and is a major cause of mortality worldwide [1–3].

Loss of muscle mass is a serious complication that affects a large proportion of patients with HF [4, 5], causing an impact on the physical capacity, quality of life, and survival of these patients. The impairment of cardiac muscle function leads to numerous neurohormonal and metabolic disorders, including an imbalance between anabolic and catabolic processes [6], being a complex scenario in which drug treatments are not available. Two distinct conditions that may be present in this group of patients, and are characterized by loss of skeletal muscle mass, are sarcopenia and cachexia.

Cachexia is a complex syndrome characterized by severe and involuntary muscle mass loss, associated or not with loss of adipose tissue. It is common in chronic diseases, such as cancer and HF, and may even occur in the absence of chronic diseases, since sarcopenia represents a slow and progressive muscle mass loss which comes with aging [7]. The prevalence of sarcopenia varies according to age groups representing 1–2% per year after 50 years, 15% at 65 years, and 50% at 80 years [8].

Regardless of its causes or mechanisms, loss of muscle mass directly contributes to exercise intolerance and impairment of daily life activities, becoming a strong predictor of reduced quality of life and mortality [9–11].

Physical exercise has been shown to be a non-pharmacological, effective, low-cost, and safe therapy that can aid in the treatment of HF, inducing anabolism through the activation or deactivation of molecular pathways essential for muscle synthesis, improving physical capacity and increasing lean mass in those patients with previous depletion [12–15]. Cardiac rehabilitation programs in patients with HF reduce mortality and hospitalization rates and improve functional ability, duration of exercise, and quality of life [16, 17].

It is well established that food consumption is a strong stimulator of muscle protein synthesis [18, 19]. Intake of protein-rich meals in the form of free amino acids [20], milk protein [21], or meat [22] is able to stimulate muscle protein synthesis. This response depends on the amount, and also on the type of protein that is ingested [23]. Whey protein (WP) is a high-quality protein that has shown superiority in enhancing muscle protein synthesis compared with other protein sources in older adults, it is rapidly digested and has a high concentration of leucine [24] that plays an important role in the stimulation of postprandial muscle protein synthesis [25]. Leucine is one of the essential amino acids in the diet. It is capable, like all branched-chain amino acids, of avoiding hepatic alterations due to the lack of L-branched chain aminotransferase in the liver and its ingestion reportedly activates the mTOR pathway in skeletal muscle, contributing to a hypertrophy response [26].

Whey protein has already demonstrated a positive effect on protein synthesis and muscle mass gain [27] when associated with exercise [28, 29], but there are no studies in the academic literature that evaluate its benefits in patients with HF.

Currently, the most promising approach to treat depletion of nutritional status due to the disease appears to be a combination therapy that includes exercise, nutritional counseling, and drug therapy [4, 30], helping to manage symptoms, reducing the number of readmissions, and improving life quality [31].

Thus, the main objective of this study will be to evaluate whether supplementation with WP alone in association with 12-week supervised exercise will be able to preserve muscle mass in patients with New York Heart Association (NYHA) functional class I or II HF. As secondary objectives, an increase in muscle strength, an improvement in endothelial function assessed through microvascular reactivity, improvement in quality of life, and reduction of inflammatory markers will be evaluated.

## Methods/design

### Study design and interventions

This is a double-blind, randomized, placebo-controlled clinical trial in which participants will be followed-up for a 12-week intervention period in which they will undergo supervised exercise through cardiac rehabilitation program participation. Participants will randomly be allocated into two groups to receive supplementation with WP alone or placebo and outcomes will be measured at baseline and upon completion of 12 weeks of study.

For the intervention group will be the supplementation of WP isolate at an amount of 30 g per day, totaling 27 g of protein and 120 kcal per serving. For the placebo group, maltodextrin will be given at an amount of 30 g per day, totaling 30 g of carbohydrates and 120 kcal per serving.

Patients will be instructed to consume supplements daily for 12 weeks, and consumption will be performed immediately after the training session on days of cardiac rehabilitation and on days without training, consumption will be performed at a time defined by the patient.

The study protocol complies with the Standard Protocol Items: Recommendations for Clinical Trials (SPIRIT) guidelines.

### Setting

The study will occur at the National Institute of Cardiology in Rio de Janeiro, Brazil, a reference unit in tertiary-level cardiology treatment linked to the Brazilian Ministry of Health.

### Participants

Patients with a clinical diagnosis of HF, with NYHA functional class I or II, and who have been referred for cardiac rehabilitation by their attending physicians, will be assessed.

### Inclusion criteria

- Age ≥ 50 years
- Heart failure functional class I or II NYHA
- Clinical stability (symptoms and medication) for more than 4 weeks
- Ejection fraction ≤ 50%

### Exclusion criteria

Creatinine clearance < 50 ml / min / 1.73 m^2^, impaired hepatic function (alanine aminotransferase > 150 U / l) or decompensated hepatic cirrhosis classified as Child-Pugh grade B or C, atrial fibrillation with heart rate > 100 bpm, allergies to milk proteins, and any osteoarticular impediment preventing physical exercise.

### Additional exclusion criteria

After the initial medical consultation, patients who are unable to understand and perform the study procedures will be excluded.

During the exercise protocol, participants will be under medical supervision to reduce any health risk, and those with decompensated heart disease will be referred to the emergency room of the hospital where the study will be conducted.

### Endpoints

The main outcome will be maintenance of muscle mass and strength. Microvascular reactivity, quality of life, and inflammatory parameters will be evaluated as secondary outcomes.

### Study procedures

**Fig. 1:**
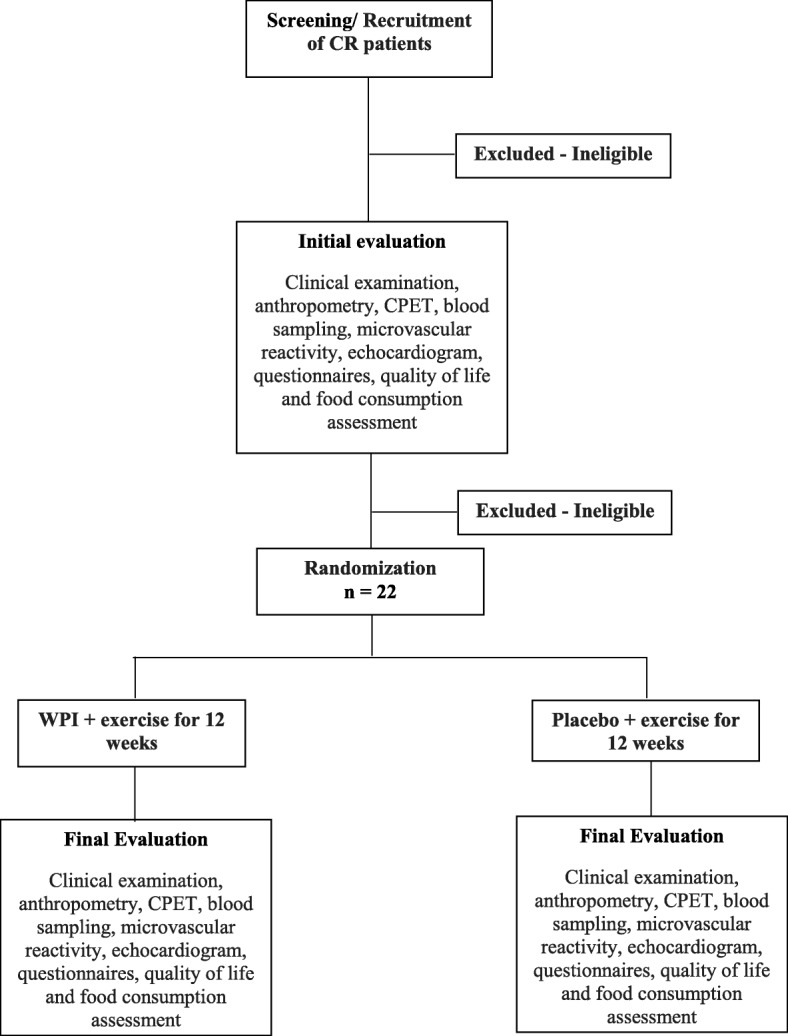
Study flowchart

Figure 1 describes all the study steps, from patient recruitment to intervention. Patients will be pre-selected from the waiting list for participation in the cardiac rehabilitation (CR) program. The waiting list consists of patients with HF who were referred to the CR program by their assisting physicians and are waiting to be scheduled. Telephone contact will be made with patients meeting the inclusion criteria, who will be scheduled for consultation with a cardiologist. During consultation, the inclusion criteria will be checked, and the informed consent will be signed. Finally, patients will be randomized and allocated to a study group.

The procedures performed at the beginning of the study will be a cardiopulmonary exercise test (CPET), echocardiogram, microvascular reactivity test, blood collection for biochemical evaluation, handgrip-strength assessment, anthropometric and body composition evaluation, health-related quality of life assessment, and assessment of dietary intake. All initial evaluations will be repeated at 12 weeks.

All staff will be trained in the operation of the equipment for data collection and obtaining written informed consent. Additionally all members of the research team will participate in the work meetings to periodically evaluate the quality of the information collected, in order to minimize errors and ensure the reliability of the data. All the research team will be trained every 6 months during the research period.

Participants will receive a weekly structured telephone interview, with questions addressing side effects (mainly gastrointestinal), symptoms, adherence, and difficulties in carrying out the guidelines provided during the study.

### Randomization and blinding

Randomization will occur in blocks of 2 and will be based on a table of random numbers generated in the online open program Openepi [32]. The random number table will be confidential. The packaging and distribution of the supplements will be performed by personnel not involved in the research. All researchers directly involved in the care and follow-up of the participating patients will not be aware of the supplements taken by the study patients. Unblinding may occur if requested by the assisting physician if a patient suffers clinical destabilization. The supplements will be delivered to the patients in metalized packages, sealed and identified by the name and code of the patient in the study, and it is not possible to identify the supplement as placebo or WP.

### Sample size

Considering a muscle mass gain of 500 g in the intervention group at 12 weeks compared to the placebo group, with a standard deviation (SD) of 409.7 g, 11 patients in each group are required to guarantee 95% confidence and 80% power to the study. The results obtained in a similar study with WP supplementation were used as a reference [33].

### Collection and storage of blood

Blood collection will be performed after 12 h of fasting. Complete blood count, fasting glucose, glycated hemoglobin, serum lipids, liver enzymes, urea, creatinine, uric acid, thyroid hormones, fibrinogen, and C-reactive protein will be analyzed.

### Nutritional assessment

Nutritional assessment will consist of anthropometric and body composition evaluations. The anthropometric evaluation will include body mass, height, and Body Mass Index calculation, waist, hip, neck, and calf-girth measurements, as well as triceps, subscapular, and calf skinfolds. All measurements will be made in duplicate and the average values ​obtained will be used.

For the evaluation of body composition, a tetrapolar eight-point tactile electrode system will be used, which separately measures impedance of the subject’s trunk, arms, and legs at six different frequencies (1 kHz, 5 kHz, 50 kHz, 250 kHz, 500 kHz, 1000 kHz) for each of the body segments. The spectrum of electrical frequencies will be used to predict the intracellular water (ICW) and extracellular water (ECW) compartments of the total body water (TBW) in the various body segments (Inbody 720®, Bioespace Co. Ltd., Seoul, South Korea). The body composition analyzer has in-built hand and foot electrodes. Subjects will wear normal indoor clothing and be advised to stand barefoot in an upright position with their feet on the foot electrodes on the machine platform and their arms abducted with hands gripping on to the hand electrodes on the handles. Subjects are require to fast for the test [34].

### Nutritional counseling

Patients will receive individualized nutritional counseling according to the treatment guidelines for HF in order to achieve adequate weight. There will be monthly consultations with a nutritionist, in which specific nutritional guidelines for each patient will be considered, according to weight, age, and gender, with calculation of caloric and protein intake and food planning.

During consultations, patients will receive supplements in individual packs labeled with name, study identification code and dose number, containing 30 g of the supplement for 4 weeks of supplementation. Patients will be advised on how to prepare the supplement and on its ingestion schedule. Each patient is expected to return the empty packages at each visit as a form of control of the supplement consumption and will be instructed not to consume other nutritional supplements during the study.

### Food consumption

Twenty-four-hour food recall will be assessed in all consultations with nutritionists. Food records will be made for 3 days a month, on two working days (one common day and 1 day with Cardiac Rehabilitation program) and one weekend day. Patients will be advised not to perform the records on consecutive days. The Food Processor® program (ESHA, Salem, OR, USA) will be used to evaluate macro and micronutrient consumption.

### Quality of life

Quality of life will be assessed using the validated Brazilian version of the Minnesota Living with Heart Failure Questionnaire (MLHFQ) [35]. The questionnaire will be applied at times T0 and T12.

### Muscle strength

The evaluation of muscle strength will be performed monthly by means of the measurement of the handgrip force using the Jamar® brand hand-held dynamometer [36]. Participants will remain seated in a standard-height chair, with a neutral adducted and rotated shoulder, elbow flexed at 90 °, forearm in neutral position, wrist in dorsiflexion of 0–30 ° and ulnar deviation of 0–15 °. The dynamometer will be adjusted based on the participant’s hand size for optimum grip position. The test will be conducted with standard verbal instructions (such as, “one, two, three, tighten ... stronger ...”) and participants will be instructed to use the non-dominant hand first, followed by the dominant hand. Three measurements will be taken for each hand, with a 10–20 s rest interval between measures to avoid fatigue. The average value of the three measures will be used [37, 38].

### Functional capacity

All patients will undergo a symptom-limited CPET, performed on a treadmill (Ergo PC Elite, Micromed, Brasilia, Brazil) with a ramp protocol. Heart rate and blood pressure will be measured every minute during exercise and recovery. A 12-lead electrocardiogram (ECG) will be continuously evaluated and will also be recorded every 3 min. After calibration of the volumes and gas exchange analyzers, patients will breathe through a mask connected to a two-way respiratory valve. Expired gases and volumes will be analyzed (VO_2000_, MedGraphics, St. Paul, MN, USA) [39].

### Echocardiogram

Conventional echocardiography will be performed in all patients using standard views, with the patient in the left lateral decubitus position, using a commercially available ultrasound machine (Vivid E9, GE Healthcare, Horten, Norway). Echocardiographic images will be obtained by a single experienced examiner and recorded for a second analysis by another experienced observer. Both will be blinded to patient allocation. Left ventricular ejection fraction (LVEF) will be calculated using the Simpson method, and all other parameters will be obtained and analyzed in accordance with the criteria defined on EAE/ASE/EACI guidelines [40, 41].

### Exercise training protocol

The exercise program will consist of supervised aerobic and strength exercises, three times a week for 12 weeks. Aerobic training will occur before strength training and will start with a 7-min warm-up at 40% of the maximal functional capacity (MFC) obtained from baseline CPET, ending with a cooling-down of 3 min at 30% of MFC (Table 1).

**Table 1:** Exercise training, prescription, and monitoring of patients on the cardiac rehabilitation program

Type of exercise		Intensity of exercise	Duration of sessions	Monitoring	Weekly frequency
Continuous exercise	Aerobic training (walking)	60–70% VO_2_max	30 min	BS (levels 12–14), BP, ECG and HR	Once a week
High-intensity interval training	Aerobic training (walking)	8 sets of 40 s at 90–95% VO_2_max separated by 110 s of moderate-intensity exercise at 50% VO_2_max	Approximately 20 min	BS (levels 12–14), BP, ECG and HR	Twice a week
Strength training	Dynamic with machine weights (quadriceps, hip, trunk, dorsal and pectoral)	3 sets of 10 repetitions (30–40% of body weight)	Approximately 30 min	Symptom-limited exercise test with HR and BP will be monitored at 10-min intervals	3 times a week

*VO*_*2*_*max* maximal oxygen uptake, *HR* heart rate, *BP* blood pressure, *BS* Borg Scale, *ECG* electrocardiogram

Once a week, patients will undergo a continuous exercise training protocol consisting of 30 min at 60–70% of MFC. Training intensity will be controlled using a heart rate monitor and the Borg Scale score aiming at levels 12–14 during the training session, as recommended in the current exercise guidelines for HF patients [16]. Twice a week, a high-intensity interval training protocol will be performed, with eight sets of 40 s at 90–95% of MFC separated by 110 s of moderate-intensity exercise at 50% of MFC. Workload will be increased during the 12-week training period based on the Borg Scale of perceived effort. The Borg Scale score and heart rate will be determined after warm-up, at 20, 30, and 40 min of exercise, and after cooling down.

Strength training will be implemented three times a week after aerobic exercise sections and will consist of eight dynamic strength exercises for larger muscles (quadriceps (2), hip (2), trunk (2), dorsal (1), and pectoral (1)) with three sets of 10 repetitions at near-maximal load separated by 1-min rest intervals between sets. During strength training, heart rate, and blood pressure will be monitored at 10-min intervals, and the load will be increased progressively once the patients can successfully perform three sets of 10 repetitions. Participants will be instructed not to perform any other exercise during the study.

### Microvascular reactivity

A laser speckle contrast imaging system with a laser wavelength of 785 nm (PeriCam PSI system, Perimed, Järfälla, Sweden) coupled to iontophoresis of acetylcholine and sodium nitroprusside will measure non-invasively real-time cutaneous microvascular flow changes in the forearm [42]. For the post-occlusive reactive hyperemia (PORH) test, arterial occlusion will be performed with suprasystolic pressure (50 mmHg above the systolic arterial pressure) using a sphygmomanometer applied to the arm of the subject over 3 min. Peak skin flow will be measured after pressure release.

Images will be analyzed using the manufacturer’s software (PIMSoft, Perimed, Järfälla, Sweden). The measurements of skin blood flow will be divided by the mean arterial pressure to yield the cutaneous vascular conductance (CVC) in arbitrary perfusion units (APU)/mmHg, to avoid interference of blood pressure levels on calculation of microvascular flow.

### Statistical analysis

Categorical variables will be presented as frequencies with percentages and will be compared using Fisher’s exact test and the chi-square test. Continuous variables will be presented as mean ± SD or median and interquartile range and will be compared using two-way analysis of variance (ANOVA) or Friedman’s test, whenever appropriate. A logistic regression analysis will be used to identify variables with an independent association with increase of lean muscle mass. A value of *p* < 0.05 will be considered statistically significant in all analyses. Calculations will be performed using the Statistical Package for the Social Sciences (SPSS 21.0 for Windows) program.

### Ethics and dissemination

The study was submitted and approved by the Ethics and Research Committee of the National Institute of Cardiology, in Rio de Janeiro, Brazil and the participants will sign the free and informed consent form. Any modifications that are made to the study will be immediately reported to the Ethics Committee responsible for approving the trial. A Data Monitoring Committee (DMC) will not be needed as this is a single-center trial. To ensure the confidentiality and the safety of participants, all information about participants will be stored safely in a computer with access restricted to the study’s investigators and, if any harm occurs, patients will have full access to free treatment at the National Institute of Cardiology, which belongs to the Brazilian public healthcare system.

Throughout the trial, the partial results will be presented at national and international scientific congresses. This study will contribute significantly to the academic training of health professionals in the area of cardiovascular sciences, committed to the production of knowledge and critical reflection, and generate new interventions capable of optimizing the treatment and quality of life of patients with HF. At the end of the study the results of the study will be published in scientific journals and presented at scientific meetings.

### SPIRIT

**Fig. 2:**
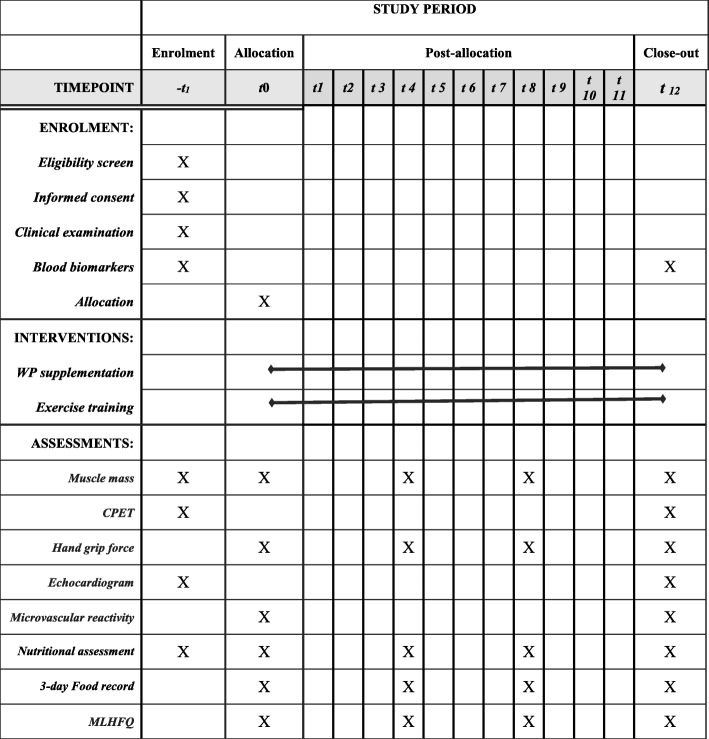
Standard Protocol Items: Recommendation for Interventional Trials (SPIRIT): the schedule of enrollment, interventions, and assessments

This protocol has been written in accordance with the Standard Protocol Items: Recommendations for Interventional Trials (SPIRIT) guidelines. The SPIRIT Checklist is in Additional file 1. The SPIRIT Figure is in Fig. 2.

## Discussion

The proposed study is a double-blind, randomized, placebo-controlled clinical trial aimed at investigating the effects of WP supplementation associated with 12 weeks of supervised exercise in maintaining muscle strength in patients with HF. Advanced HF is often associated with severe loss of body weight and especially of muscle mass, and it is an important cause of hospitalization and one of the most important health challenges since its prevalence tends to increase with the aging of the population and the increase in the survival of patients who have suffered acute coronary events. It is an ongoing epidemic problem, resulting in a high socioeconomic cost, represented by the expenditure on medicines, repeated hospitalizations, loss of productivity, early retirement, possible surgeries and, ultimately, the need for heart transplantation. In this sense, it is of great importance to carry out studies that evaluate the possible benefits of new clinical and nutritional interventions for HF patients, favoring the development of new treatment strategies.

### Trial status

Patient recruitment is currently being undertaken.

## Additional file

Additional file 1: Standard Protocol Items: Recommendations for Interventional Trials (SPIRIT)_guidelines_WPROT_HF. (PDF 208 kb)

## Abbreviations

APU: Arbitrary perfusion units
CPET: Cardiopulmonary exercise test
CR: Cardiac rehabilitation
CVC: Cutaneous vascular conductance
DMC: Data Monitoring Committee
ECG: Electrocardiogram
ECW: Extracellular water
HF: Heart failure
ICW: Intracellular water
LVEF: Left ventricular ejection fraction
MFC: Maximal functional capacity
MLHFQ: Minnesota Living with Heart Failure Questionnaire
NYHA: New York Heart Association
PORH: Post-occlusive reactive hyperemia
SPSS: Statistical Package for the Social Sciences
TBW: Total body water
WP: Whey protein
